# Interactions between ethylene and auxin are crucial to the control of grape (*Vitis vinifera* L.) berry ripening

**DOI:** 10.1186/1471-2229-13-222

**Published:** 2013-12-23

**Authors:** Christine Böttcher, Crista A Burbidge, Paul K Boss, Christopher Davies

**Affiliations:** 1 CSIRO Plant Industry, PO Box 350, Glen Osmond, SA 5064, Australia

**Keywords:** Aminoethoxyvinylglycine, Auxin, Biosynthesis, Ethrel, Ethylene, Interaction, *Vitis vinifera*, Ripening

## Abstract

**Background:**

Fruit development is controlled by plant hormones, but the role of hormone interactions during fruit ripening is poorly understood. Interactions between ethylene and the auxin indole-3-acetic acid (IAA) are likely to be crucial during the ripening process, since both hormones have been shown to be implicated in the control of ripening in a range of different fruit species.

**Results:**

Grapevine (*Vitis vinifera* L.) homologues of the TRYPTOPHAN AMINOTRANSFERASE RELATED (TAR) and YUCCA families, functioning in the only characterized pathway of auxin biosynthesis, were identified and the expression of several *TAR* genes was shown to be induced by the pre-ripening application of the ethylene-releasing compound Ethrel. The induction of *TAR* expression was accompanied by increased IAA and IAA-Asp concentrations, indicative of an upregulation of auxin biosynthesis and conjugation. Exposure of *ex planta,* pre-ripening berries to the ethylene biosynthesis inhibitor aminoethoxyvinylglycine resulted in decreased IAA and IAA-Asp concentrations. The delayed initiation of ripening observed in Ethrel-treated berries might therefore represent an indirect ethylene effect mediated by increased auxin concentrations. During berry development, the expression of three *TAR* genes and one *YUCCA* gene was upregulated at the time of ripening initiation and/or during ripening. This increase in auxin biosynthesis gene expression was preceded by high expression levels of the ethylene biosynthesis genes 1-aminocyclopropane-1-carboxylate synthase and 1-aminocyclopropane-1-carboxylate oxidase.

**Conclusions:**

In grape berries, members of both gene families involved in the two-step pathway of auxin biosynthesis are expressed, suggesting that IAA is produced through the combined action of TAR and YUCCA proteins in developing berries. The induction of *TAR* expression by Ethrel applications and the developmental expression patterns of auxin and ethylene biosynthesis genes indicate that elevated concentrations of ethylene prior to the initiation of ripening might lead to an increased production of IAA, suggesting a complex involvement of this auxin and its conjugates in grape berry ripening.

## Background

The coordinated growth and development of plants relies on a wide range of control systems, including an intricate network of interactions between all classes of known plant hormones and other signalling compounds such as sugars [1-5]. Fruit ripening is an example of a developmentally controlled process that is dependent on a range of plant hormones, but our knowledge about the molecular interactions of these different classes of hormones in the ripening process is still rudimentary (reviewed by [6,7]). An interesting target for the investigation of hormonal interplay in fruit is the interaction between the gaseous hormone ethylene and the auxin class of hormones, of which indole-3-acetic acid (IAA) is the most abundant member. Ethylene plays a crucial role in the ripening of climacteric fruit which display a sharp increase in ethylene production and respiratory activity at the onset of ripening (reviewed by [8]), but its relevance for the ripening of non-climacteric fruit, which lack the dramatic, ripening-related change in ethylene formation and respiration, is still unclear (reviewed by [6]). A moderate increase in ethylene concentration and respiration rate has been demonstrated for the non-climacteric strawberry (*Fragaria ananassa* Duch.) [9], but both events occurred after the transition into the ripening phase. The only reports of small peaks in ethylene concentration just before the initiation of ripening in non-climacteric fruit come from *Litchi chinensis* Sonn. [10] and grape (*Vitis vinifera* L.) [11-13]. In grape, a role for ethylene is further evidenced by the developmental expression profiles of genes encoding for the ethylene biosynthesis enzymes 1-aminocyclopropane-1-carboxylate synthase (ACS) and 1-aminocyclopropane-1-carboxylate oxidase (ACO), which describe a pre-ripening expression for *ACS*[14] and a peak in transcript accumulation for *ACO*[12,14] a short time before the initiation of ripening (called ‘veraison’ in grape berries).

Interestingly, in contrast to what is known from climacteric fruit, the treatment of pre-veraison grape berries with the ethylene-releasing compound Ethrel has been shown to delay ripening [15-17]. The Ethrel-induced delay in the onset of sugar and anthocyanin accumulation resembles the ripening-delaying effects caused by the pre-veraison application of auxins [18-23], which have emerged as another important factor in fruit ripening in both climacteric and non-climacteric fruit (reviewed by [6,7]). In climacteric fruit, such as tomato (*Solanum lycopersicum* Mill.) [24,25], or banana (*Musa paradisiaca* L.) [26] and in non-climacteric fruit like strawberry [27,28] and grape [29-32] the concentrations of IAA before and during ripening were generally found to be low. In all four fruit species mentioned above, as well as in many other climacteric and non-climacteric fruit, the application of natural or synthetic auxins during the pre-ripening stage of fruit development has often been found to lead to a ripening delay (reviewed by [6]) and therefore auxins are widely viewed as ripening inhibitors [24,26,29,33]. In contrast IAA-amide conjugates have been reported to accumulate in ripening bananas [26], muskmelons (*Cucumis melo* L.) [34] and strawberries [27]. More detailed studies in grape berries have revealed that the concentration of IAA-Asp, an IAA-amide conjugate linked to IAA degradation [35] and formed by the action of IAA-amido synthetases (Gretchen Hagen (GH3) proteins) [36], increased sharply at veraison and remained at high concentrations throughout the ripening phase [29]. A similar pattern of IAA-Asp accumulation has been found in tomato which suggests a more complex role for auxins in fruit ripening that requires further investigation [29].

The best understood interaction between auxin and ethylene is the induction of ethylene biosynthesis by applied auxins, which was first described by Morgan and Hall [37] who reported that 2,4-dichlorophenoxyacetic acid application led to an increase in ethylene production in cotton plants. It was later revealed that exogenous auxins induce the transcription of *ACS* genes in mung beans (*Vigna radiata* (L.) Wilczek) [38]. A similar induction of *ACS* expression and in some instances also *ACO* expression has since been demonstrated for many other plant species, including *Arabidopsis* (*Arabidopsis thaliana* L.) [39-41], a range of climacteric and non-climacteric fruit such as apple (*Malus domestica* Borkh.) [42], pear (*Pyrus communis* L.) [43], peach (*Prunus persica* (L.) Batsch) [44] and grape [45]. Reports about an acceleration of at least parts of the ripening process by auxin treatments in some climacteric fruit [46-50] can therefore be interpreted as an indirect auxin effect mediated through the induction of ethylene biosynthesis [42,44,48].

The complementary effect of ethylene on auxin biosynthesis has long remained elusive, due to an incomplete understanding of the molecular mechanisms involved in the biosynthesis of IAA. A dependence of ethylene action on IAA biosynthesis, perception, signalling and transport has been illustrated in studies using *Arabidopsis* mutants and auxin measurements [51,52]. Furthermore, the induction of genes encoding for anthranilate synthase and tryptophan synthase subunits as well as a GH3 protein by ethylene treatments has been reported [53,54]. However, only the recent elucidation of the main IAA biosynthesis pathway in *Arabidopsis* has revealed the full extent of the regulation of auxin biosynthesis by ethylene and the dependence of ethylene effects on auxin production. Three independent studies [55-57] have provided evidence that IAA is synthesised via a simple two-step pathway in which the family of TRYPTOPHAN AMINOTRANSFERASE OF ARABIDOPSIS1/TRYPTOPHAN AMINOTRANSFERASE RELATED (TAA1/TAR) proteins [58,59] converts tryptophan to indole-3-pyruvate which is then converted to IAA by the YUCCA (YUC) family of flavin-containing monooxygenases [60]. The existence of a TAR/YUC pathway has also been confirmed in maize (*Zea mays* L.) where it is essential for normal vegetative and reproductive development [61]. The expression of *TAA1* as well as some *TAR*s in roots and apical hooks of *Arabidopsis* seedlings can be induced by ethylene treatments and the restricted expression of *TAA1/TAR* genes leading to local IAA production is thought to be the basis of tissue-specific ethylene effects [58].

The above introduction shows that auxins and ethylene are involved in berry ripening and that their application can alter the progression of ripening. In this study, evidence is provided for a functional TAR/YUC pathway of auxin biosynthesis in developing grape berries which is at least in part regulated by ethylene. Auxin measurements as well as gene expression studies in Ethrel-treated fruit suggest that the Ethrel-induced ripening delay in grapes might be mediated by increased auxin concentrations in pre-ripening fruit. In developing berries an increase in the expression of ethylene biosynthesis genes precedes elevated transcript levels of auxin biosynthesis and conjugation genes at around the initiation of ripening, indicative of a role for ethylene/auxin interactions in the control of berry ripening.

## Results and discussion

### Pre-veraison Ethrel treatment of grape berries leads to ripening delay and increased auxin concentrations

**Figure 1:**
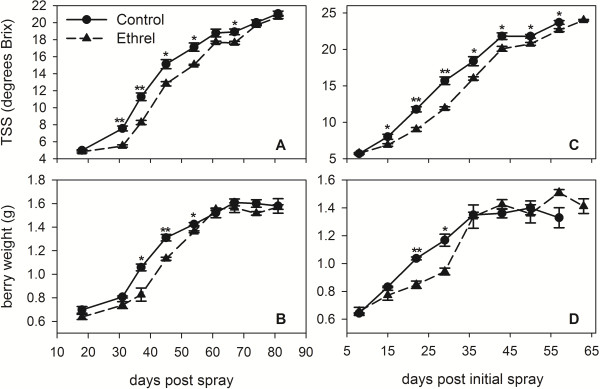
**Delayed ripening of Shiraz berries after treatment with Ethrel.** Changes in TSS, measured as degrees Brix, in field grown Shiraz berries treated **(A)** once in 2011 (20 days pre-veraison) or **(C)** twice in 2012 (8 and 1 days pre-veraison) with 300 μL L^-1^ Ethrel in 0.1% (v/v) Chemwet 1000 or 0.1% (v/v) Chemwet 1000 (Control). The same berry samples were used to measure changes in berry weight in **(B)** the 2011 and **(D)** the 2012 samples. Control, closed circles with solid lines; Ethrel, closed triangles with dashed line. All data represent means ± SE (n = 3). Asterisks indicate significant differences of the mean values of Ethrel-treated samples from the mean values of Control samples as determined with Student’s *t*-test (*p < 0.05, **p < 0.01).

In grape berries, the initiation of ripening can be delayed by the pre-veraison application of the ethylene-releasing compound Ethrel [15-17] and auxins [18-23]. The similar effects of Ethrel and auxins on the ripening behaviour of grapes combined with the increasing evidence for complex interactions between ethylene and IAA (reviewed by [62,63]) is suggestive of a link between these two plant hormones in the control of ripening. In order to investigate the possibility of an ethylene/auxin interaction in grape berry ripening, pre-veraison Shiraz berries were treated with either Ethrel or a Control solution in two consecutive seasons. In the 2011 trial, the Ethrel treatment of berries 20 days pre-veraison (veraison is defined as the sampling date immediately prior to a significant increase in total soluble solids (TSS) measured using a refractometer) delayed the onset of ripening by 13 days (veraison of Control berries was 3 February 2011). The Ethrel-treated berries had significantly lower TSS values between 31 and 54 days post spray (dps) and at 67 dps when compared with the Control berries (Figure 1A) and there was also a reduction in berry weight between 37 and 54 dps (Figure 1B).

In 2012, veraison of the Control berries (24 January 2012) occurred one week earlier than predicted, which brought the two successive treatments that were applied during that season close to the veraison date (eight and one day prior to veraison). In previous studies, Ethrel-treatments within a week of veraison had no effect on sugar accumulation and berry weight [15]. Accordingly, the ripening-delaying effect of Ethrel in the 2012 trial was less pronounced than what had been observed in 2011. Veraison of the Ethrel-treated berries was only delayed by seven days. However, from 15 days post initial spray (dpis) onwards the TSS values in the Ethrel-treated berries were significantly lower when compared with the Control berries (Figure 1C) and a difference in berry weight was observed 22 and 29 dpis when the Ethrel-treated berries were significantly lighter (Figure 1D).

A time course of berry samples from both trials was used to determine if the Ethrel treatments had caused changes in the concentrations of IAA and its most abundant amino acid conjugate, IAA-Asp [29]. For the 2011 experiment this was done 3–48 hours post spray (hps), which was extended for the 2012 trial to test for differences in auxin concentrations from one hour post initial spray (hpis) through to the time point of veraison of the Ethrel-treated berries.

**Figure 2:**
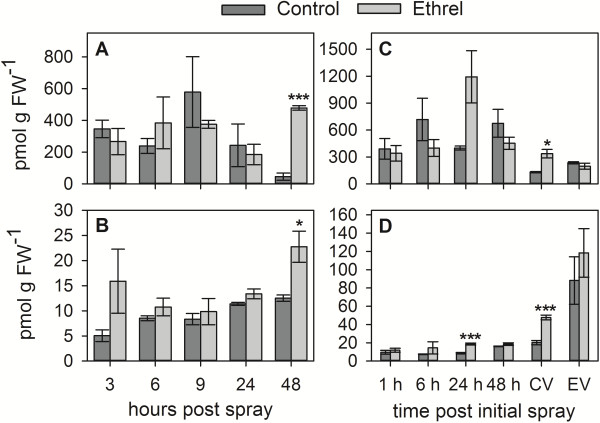
**Changes in the concentration of IAA and IAA-Asp in pre-veraison berries in response to Ethrel application.** IAA **(A, C)** and the IAA-amino acid conjugate, IAA-Asp, **(B, D)** were quantified by LC-MS/MS in pre-veraison Shiraz berries from the 2011 trial **(A, B)** and the 2012 trial **(C, D)** at the indicated time points after treatment with a Control or Ethrel solution as described in Figure 1. Control, dark grey bars; Ethrel, light grey bars. FW, fresh weight. CV, veraison of Control fruit; EV, veraison of Ethrel-treated fruit. All data represent means ± SE (n = 3). Asterisks indicate significant differences of the mean values of Ethrel-treated samples from the mean values of Control samples as determined with Student’s *t*-test (*p < 0.05, ***p < 0.001).

When compared with the Control fruit at each time point, IAA and IAA-Asp concentrations in the 2011 experiment were only significantly different from the Ethrel-treated berries at the last time point analysed (48 hps), where the IAA concentration was increased approximately ten-fold (Figure 2A) and the IAA-Asp concentration approximately two-fold (Figure 2B) in response to Ethrel. In the 2012 samples the IAA concentration was significantly higher in the Ethrel-treated berries when the Control berries were going through veraison (~ three-fold) (Figure 2C) and IAA-Asp concentrations were increased by Ethrel about two-fold 24 hpis and at veraison of the Control berries (Figure 2D).

The presented data provide evidence for an Ethrel-mediated increase in the accumulation of free and conjugated IAA in pre-veraison grape berries. The lasting elevation of auxin concentrations up to the transition into the ripening phase poses the question whether the Ethrel-induced ripening delay might be the result of higher than normal auxin concentrations in pre-veraison berries.

### IAA and IAA-Asp levels and grape homologues of auxin biosynthesis genes are upregulated in berries by Ethrel while auxin levels are repressed by aminoethoxyvinylglycine

**Figure 3:**
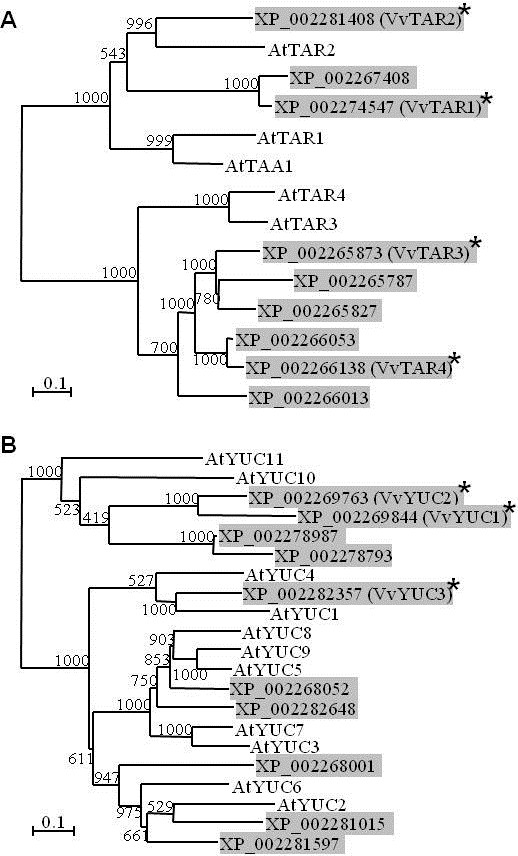
**Phylogenetic relationship of TAR and YUC protein sequences from grapevine and *Arabidopsis*.** Unrooted trees of **(A)** TAR sequences and **(B)** YUC sequences were generated with the PHYLIP program [81] using the neighbour-joining method and a bootstrap test with 1000 iterations (bootstrap values are indicated at each node). The scale bar indicates genetic distance based on branch length. The predicted grapevine proteins are highlighted with a shaded background. Asterisks indicate grapevine sequences with EST entries in the NCBI database. At, *Arabidopsis thaliana*; Vv, *Vitis vinifera*. Accession numbers of the *Arabidopsis* protein sequences used in this analysis are provided in Additional file 3.

Recent studies on *Arabidopsis* seedlings have provided evidence that ethylene is involved in the establishment of local auxin maxima by inducing the expression of *TAA1* and related genes (*TAR*s) [58]. Together with YUC proteins TAA1/TARs are a component of the only complete pathway of auxin biosynthesis in plants described to date [55-57]. In order to investigate if the increase in auxin concentrations in Ethrel-treated grape berries might be due to a stimulation of the TAR/YUC pathway, grapevine homologues of the *Arabidopsis* TAA1 and YUC1 proteins were identified through a BLASTP similarity search on the NCBI database. The resulting nine TAR and ten YUC grapevine sequences were aligned to the respective protein families from *Arabidopsis* and unrooted phylogenetic trees were constructed from the alignments (Figure 3A, B). Any further analyses were restricted to those members of the grapevine families that were verified as being expressed in any tissues by the presence of ESTs in the NCBI database. These were VvTAR1 (55% identity with AtTAA1), VvTAR2 (59% identity with AtTAR2), VvTAR3 (53% identity with AtTAR3) and VvTAR4 (55% identity with AtTAR3) (Figure 3A), as well as VvYUC1 (49% identity with AtYUC10), VvYUC2 (51% identity with AtYUC10) and VvYUC3 (73% identity with AtYUC4) (Figure 3B). All attempts to amplify *VvYUC2* fragments from a number of different grapevine cDNAs (from flowers, berries at different stages, roots, leaves) for the generation of qRT-PCR standards failed (data not shown), so this gene could not be included in expression analyses.

The expression of the putative auxin biosynthesis genes from the *TAR* and *YUC* families, as well as the previously described grapevine *GH3* genes (*GH3-1* and *GH3-2*) involved in IAA-Asp formation in berries [19,29,64], was analysed in the same samples that had been used for the quantitation of IAA and IAA-Asp (see above, Figure 2A-D). To verify the activation of an ethylene response in the Ethrel-treated berries, the expression of the ethylene biosynthesis genes *ACS1* and *ACO1* as well as the expression of the ethylene receptor gene *ETR2* was also analysed. In a previous study on grape berries, *ACS1* expression was not changed by Ethrel treatments three weeks to one week prior to veraison, whereas the expression of *ACO1* and *ETR2* was Ethrel-inducible between three and two weeks pre-veraison [15]. *ETR2* is commonly used as a marker for ethylene responses that has been employed in research on vegetative tissues [65] as well as fruit [66].

**Figure 4:**
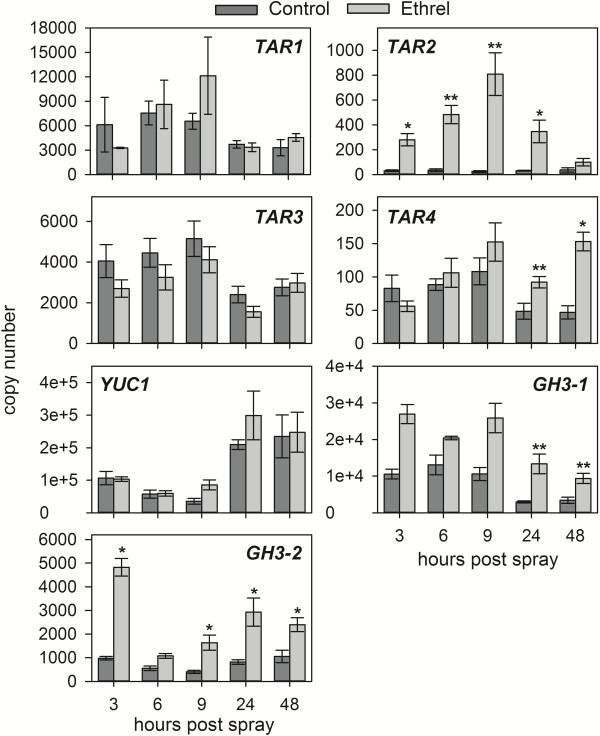
**Transcription of selected auxin biosynthesis and *GH3 *genes in response to Ethrel in the 2011 trial.** The expression of *TAR1-TAR4*, *YUC1*, *GH3-1* and *GH3-2* in pre-veraison Shiraz berries from the 2011 trial was analysed by qRT-PCR at the indicated time points after treatment with a Control or Ethrel solution (see Figure 1). Control, dark grey bars; Ethrel, light grey bars. All data represent means ± SE (n = 3). Asterisks indicate significant differences of the mean values of Ethrel-treated samples from the mean values of Control samples as determined with Student’s *t*-test (*p < 0.05, **p < 0.01).

In the 2011 experiment, a strong response to Ethrel was demonstrated by the induction of *ETR2* expression, by up to 20-fold, 3–48 h after the Ethrel spray (Additional file 1A). The expression of *ACS1* (9 and 24 hps) and *ACO1* (9–48 hps) was also upregulated (two to 13-fold) (Additional file 1A). The expression of *YUC3* was not detectable in any of the samples analysed. The transcription of *TAR2* was increased up to 30-fold in Ethrel-treated berries between 3–24 hps (Figure 4). There was also an induction (two to three-fold) of *TAR4* at 24 hps and 48 hps, whereas the expression of *TAR1* and *TAR3* was not significantly different between the Control and Ethrel samples at all time points (Figure 4). As for *TAR1* and *TAR3* there was no significant difference in the expression of *YUC1* at any of the tested time points between the Ethrel-treated and the Control berries (Figure 4). Similar to *TAR4*, *GH3-1* expression was significantly upregulated by the Ethrel treatment after 24 h and 48 h (up to 4.5-fold). The transcription of *GH3-2* was also induced at 3 hps (five-fold) and at 9–48 hps (up to four-fold) (Figure 4). The increase in *GH3* expression as well as *TAR4* expression correlated with the higher IAA-Asp and IAA concentrations in Ethrel-treated berries 48 hps (Figure 2A, B). The highly elevated expression of *TAR2* preceded the increase in free IAA and conjugate concentrations after 48 h. This could be due to a lag between the increase in transcript levels and the production of active enzyme, but it might also reflect break down of IAA and/or IAA-Asp, sequestration of IAA into conjugates other than IAA-Asp or transport of auxins out of the berry.

**Figure 5:**
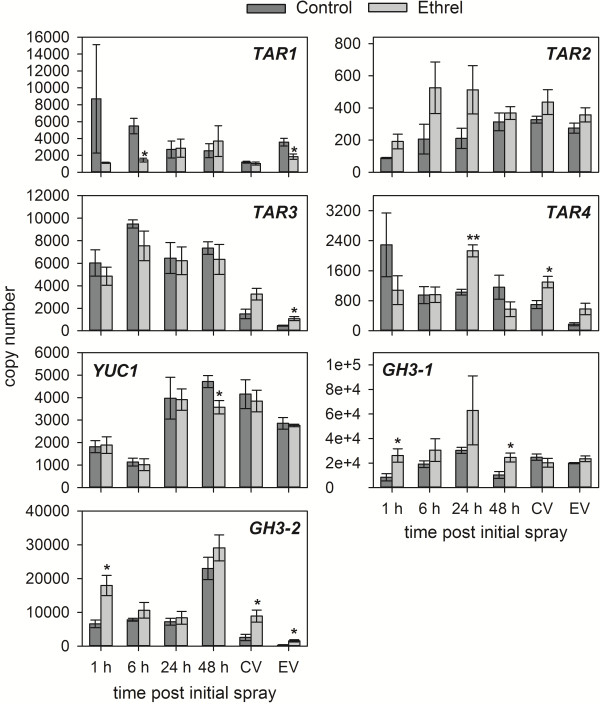
**Changes in transcript accumulation of selected auxin biosynthesis and *GH3 *genes in response to Ethrel in the 2012 trial.** The expression of *TAR1-TAR4*, *YUC1*, *GH3-1* and *GH3-2* in pre-veraison Shiraz berries from the 2012 trial was analysed by qRT-PCR at the indicated time points after treatment with a Control or Ethrel solution (see Figure 1). Control, dark grey bars; Ethrel, light grey bars. CV, veraison of Control fruit; EV, veraison of Ethrel-treated fruit. All data represent means ± SE (n = 3). Asterisks indicate significant differences of the mean values of Ethrel-treated samples from the mean values of Control samples as determined with Student’s *t*-test (*p < 0.05, **p < 0.01).

In the 2012 experiment, where only a minor delay in ripening of the Ethrel-treated berries was observed, the expression analysis of *ACS1*, *ACO1* and *ETR2* also reflected a weak response to Ethrel (Additional file 1B). All three genes were only found to have a significant increase in transcription at one time point. Accordingly, the changes in expression of the IAA biosynthesis and conjugation genes were also less pronounced than in the previous season. As in 2011, *TAR1* expression was similar in Control berries and Ethrel-treated fruit. However, there was a significant reduction in *TAR1* transcript abundance in Ethrel-treated berries 6 hpis and at the time of veraison of the Ethrel-treated fruit (Figure 5). There was no significant change in expression of *TAR2* between Ethrel-treated berries and Control berries (Figure 5). There were two-times more *TAR3* transcripts in Ethrel-treated fruit as the berries were going through veraison and the expression of *TAR4* was increased by the Ethrel treatment 24 hpis and at the time point of veraison of the Control berries (two-fold) (Figure 5). The elevated expression of *TAR4* coincided with the slight increase in free IAA concentration at veraison of the Control berries (Figure 2C). *YUC1* expression was reduced by the Ethrel treatment 48 hpis (Figure 5) and *YUC3* expression could not be detected. The expression of both *GH3* genes was increased by about three-fold 1 h after the initial Ethrel spray. There was also an induction of *GH3-1* expression 48 hpis and the transcript accumulation of *GH3-2* was increased in the Ethrel-treated berries at the time of veraison in the Control and the Ethrel-treated fruit (Figure 5).

**Figure 6:**
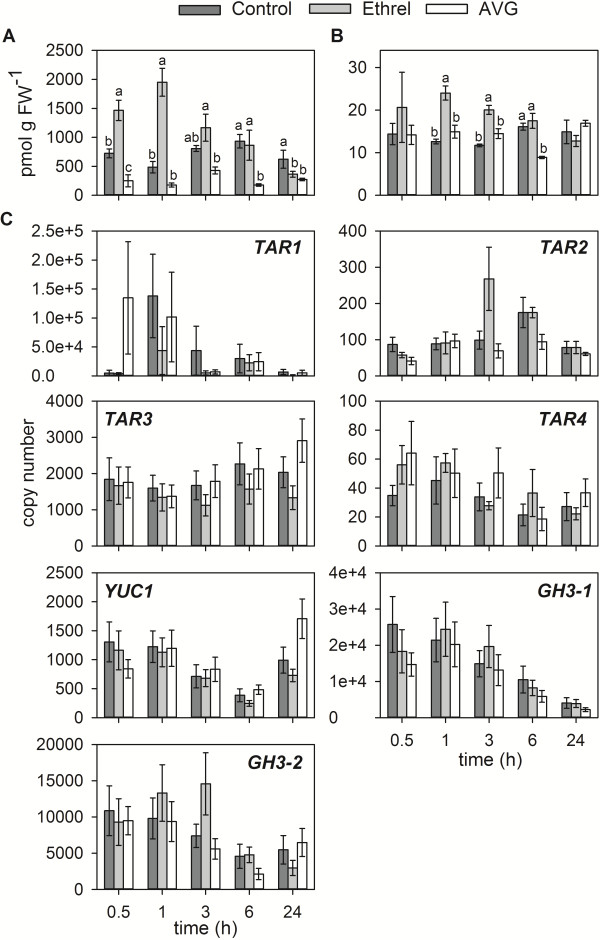
**Effect of Ethrel and AVG on auxin concentrations and the expression of selected auxin biosynthesis and *GH3 *genes in an *ex planta *experiment.** IAA **(A)** and the IAA-amino acid conjugate, IAA-Asp, **(B)** were quantified by LC-MS/MS in *ex planta* pre-veraison Shiraz berries exposed to ReTain (125 mg L^-1^ AVG, 3% (w/v) sucrose), Ethrel (72 mg L^-1^ ethephon, 3% (w/v) sucrose), or Control (3% (w/v) sucrose) conditions for the indicated periods of time. FW, fresh weight. **(C)** Using the same tissues the expression of *TAR1-TAR4*, *YUC1*, *GH3-1* and *GH3-2* was analysed by qRT-PCR. Control, dark grey bars; Ethrel, light grey bars; AVG, white bars. Bars represent means ± SE (n = 3) and are denoted by a different letter if the means for each time point differ significantly (p < 0.05) using one-way ANOVA followed by Duncan’s post hoc test.

In a recent study on the effects of ethylene on grape berry ripening, it was found that aminoethoxyvinylglycine (AVG), an inhibitor of ethylene biosynthesis, had a ripening-advancing effect on berries when applied between three to one weeks prior to veraison [15]. AVG has long been known to be a competitive inhibitor of ACS proteins [67,68], thereby blocking the conversion of S-adenosyl-methionine to 1-aminocyclopropane-1-carboxylic acid [69]. When Capitani et al. [70] solved the crystal structure of an apple ACS protein, it was revealed that the active site of this enzyme is highly similar to that of the related family of aminotransferases [71]. In accordance with this finding, AVG has recently been described as an inhibitor of auxin biosynthesis in *Arabidopsis* with the TAA1/TAR family as its target [72]. Independent of ethylene, AVG treatments led to a rapid reduction in the concentration of free IAA in *Arabidopsis* seedlings and in leaves of tomato and tobacco (*Nicotiana tabacum* L.) plants in a dose-dependent manner. The results of this study confirmed earlier reports about a 50-60% reduction in the rate of IAA biosynthesis in *Arabidopsis* seedlings exposed to AVG [52]. Based on these recent data on AVG action, the effect of AVG on auxin production in pre-veraison Shiraz berries was investigated in an *ex planta* experiment. Berries were exposed to AVG, Ethrel and a Control treatment for 0.5-24 h followed by the extraction and quantitation of IAA and IAA-Asp. Already at the first time point of analysis (0.5 h after the initiation of treatment) a two-fold increase in the concentration of IAA was found in the Ethrel-treated berries, whereas a three-fold reduction in the concentration of IAA was measured in AVG-treated fruit (Figure 6A). The Ethrel-induced increase in IAA accumulation was transient and only lasted for 1 h after which the IAA concentration returned to levels observed in the Control and even slightly decreased after 24 h of exposure. However, AVG caused a significant reduction in IAA concentrations after 6–24 h of exposure (Figure 6A). A similar, but delayed, trend was observed for changes in the IAA-Asp concentrations (Figure 6B). Exposure to Ethrel resulted in a significant increase in IAA-Asp accumulation after 1 h and 3 h, whereas the treatment with AVG led to a reduced IAA-Asp concentration after 6 h (two-fold). The rapid changes in auxin concentrations in response to Ethrel and AVG in the *ex planta* experiment when compared with the data obtained from berries that were sprayed in the field (see above, Figure 2) is most likely due to a higher uptake rate of the compounds under the *ex planta* conditions and to continuous exposure to high levels. It can therefore be inferred that changes in auxin biosynthesis and conjugation occurred within the first 30 min of exposure, which explains the unchanged expression of the *TAR*, *YUC* and *GH3* genes analysed between 0.5 and 24 h of exposure (Figure 6C). There was also no difference in the expression of *ACS1* and *ACO1* between Control, Ethrel-treated and AVG-treated berries (Additional file 1C). The only significant change in *ETR2* expression occurred at 3 h after Ethrel exposure (two-fold increase) (Additional file 1C). A repression of *ACO1* and *ETR2* expression by AVG after 20 h of exposure has been reported from a similar grape berry *ex planta* experiment [15]. Since both these genes are sucrose-responsive [15], this apparently conflicting result is most likely due to the different sucrose concentrations used in the previous study (0% or 12% (w/v)) compared with this study (3% (w/v)).

It can be concluded that the TAR/YUC pathway of auxin biosynthesis is active in grape berries and can be stimulated by increased concentrations of ethylene. As a consequence, ripening-delay by Ethrel may be mediated by elevated auxin formation in the berry tissue. More studies are needed to determine if the advancement of berry ripening by AVG applications is the result of decreased ethylene or auxin concentrations or possibly of both.

### Changes in the expression of auxin and ethylene biosynthesis genes during development suggest a role for both hormones in the initiation of grape berry ripening

**Figure 7:**
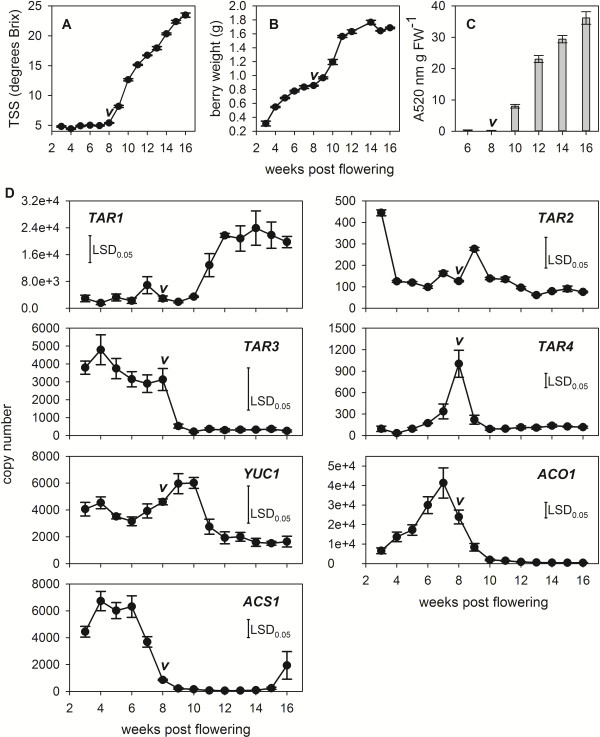
**Expression profiles of selected auxin and ethylene biosynthesis genes throughout Shiraz berry development.** The development of field-grown Shiraz berries was documented by changes in **(A)** TSS, **(B)** berry weight and **(C)** anthocyanin (A520 nm) accumulation. **(D)** Between 3–16 wpf the expression of *TAR1-TAR4*, *YUC1*, *ACS1* and *ACO1* was analysed by qRT-PCR. “*v*” indicates veraison as determined by the last time point before a significant increase (ANOVA followed by Duncan’s post hoc test) in TSS levels was recorded. All data represent means ± SE (n = 3) and for the gene expression data LSD values were determined at the p < 0.05 significance level.

Previous studies on grape berries as well as other non-climacteric fruit have reported low concentrations of free IAA and ethylene at the time of ripening initiation (reviewed by [6]). However, a subtle increase in ethylene concentrations coinciding with the beginning of the ripening phase in *L. chinensis* and grapes [10-13], as well as a drastic increase in the accumulation of IAA-Asp in ripening grapes and tomatoes [29], suggest that both hormones might be of importance for the transition from the pre-ripening to the ripening stage. To further investigate the implications of ethylene and auxin in grape berry ripening, the expression of *TAR1-4*, *YUC1*, *YUC3* as well as *ACS1* and *ACO1* was analysed in Shiraz berries from 3 weeks post flowering (wpf) through to harvest at 16 wpf. Veraison, defined as the last time point before a significant increase in TSS levels was observed, was found to be 8 wpf (Figure 7A). It also marked the start of the second growth phase and the accumulation of anthocyanins (Figure 7B, C). *TAR1* was expressed at low levels in pre-veraison berries, but copy numbers increased rapidly between 10–12 wpf and remained high through to harvest after 16 weeks (Figure 7D). *TAR3* was expressed in pre-veraison berries, whereas the expression profiles of *TAR2* and *TAR4* were characterized by a significant peak in transcript levels at veraison (*TAR4*) or one week post-veraison (*TAR2*) (Figure 7D). A comparable peak in expression was also observed for *YUC1* (8–10 wpf) (Figure 7D), whereas *YUC3* was found to be expressed at very low levels in young berries (one and two wpf), but *YUC3* transcripts could not be reliably detected in berries 3–16 wpf (data not shown). An increase in the expression of *YUC* genes, similar to *YUC1*, has recently been reported in strawberries, where the transcript accumulation of Fa*YUC1* and Fa*YUC2* in achenes peaked at the large green fruit stage [73]. In grape berries, the increase in expression of the putative auxin-biosynthesis genes was preceded by high expression levels of the ethylene biosynthesis genes *ACS1* (3–7 wpf) and *ACO1* (peaking at 7 wpf) (Figure 7D) which confirmed previous results for the expression of *ACS/ACO* in developing grape berries [12,14,15]. Similar expression patterns of all eight genes were recorded from a 3–16 wpf berry series from Cabernet Sauvignon (Additional file 2).

The data suggest that a peak in auxin biosynthesis coincides with the initiation of ripening in grape berries and that this elevated auxin production might be induced by ethylene. The post-veraison expression of *TAR1* further indicates that IAA is synthesized throughout the ripening period. An increase in IAA biosynthesis at and after veraison seems to contradict the low concentrations of this auxin in ripening berries as well as the ripening-delaying effects of high levels of auxins in pre-veraison berries. However, the sharp increase in IAA-Asp accumulation just after veraison reported by Böttcher et al. [29] might indicate that all IAA produced during a short period around the start of ripening and possibly throughout ripening is sequestered via the conjugation pathway. This proposal is supported by the increased expression of *GH3-1* during this time in berry development. Furthermore, in tomatoes, which also display increased concentrations of IAA-Asp during ripening [29], the overexpression of a pepper (*Capsicum chinense* L.) *GH3* gene stimulated an early transition into the ripening phase upon an ethylene stimulus [74] and the RNAi suppression of *APETALA2*, a negative regulator of ripening [75], resulted in earlier ripening and an increased accumulation of *GH3* transcripts [76]. The long-held view that IAA-amino acid conjugates are merely means to store or degrade the free auxin [77] has been challenged by a number of studies that provide evidence for an active role of IAA-Asp and other conjugates in plant development and disease response (reviewed by [78]). It is therefore conceivable that the IAA-Asp conjugate might represent a ripening signal in grapes, and possibly also in other fruit species, which can be perceived at a certain developmental stage of the fruit by an as yet unknown mechanism. From the presented data, a possible role of ethylene in the ripening of grape berries could be the triggering of IAA synthesis and subsequent IAA-Asp formation at the above mentioned critical developmental stage. Removal of the ethylene trigger should consequently lead to ripening inhibition. This hypothesis is supported by studies from Chervin et al. [12,79], where blocking of ethylene perception close to veraison led to reduced berry size and delayed anthocyanin and sugar accumulation.

## Conclusions

Despite an increasing knowledge of the involvement of ethylene and IAA in fruit development, information about interactions between these two hormones in fruit is limited. The present study provides evidence of a functional two-step pathway of auxin biosynthesis in grape berries and demonstrates its activation by the ethylene-releasing compound Ethrel. An observed ripening delay in Ethrel-treated fruit might therefore be due to increased auxin concentrations in pre-ripening fruit. The expression patterns of auxin and ethylene biosynthesis genes during berry development further suggest that an increase in ethylene production prior to the initiation of ripening stimulates IAA biosynthesis at the commencement of ripening. This developmentally controlled induction of auxin biosynthesis by ethylene might be required for the rapid accumulation of the proposed ripening factor, IAA-Asp.

## Methods

### Plant material

Ethrel treatments were performed using *Vitis vinifera* L. cv Shiraz fruit in two consecutive years at the same site (Hahndorf, South Australia, -35.018223, 138.838220). In 2011, pre-veraison berries were sprayed to run off once (14 January 2011, 20 days pre-veraison) with 300 μL L^-1^ Ethrel (144 mg L^-1^ ethephon; Bayer CropScience, East Hawthorn, Australia) in 0.1% (v/v) Chemwet 1000 (Nufarm, Laverton, Australia), solution pH 3.1. Control fruit were sprayed with a 0.1% (v/v) Chemwet 1000 solution. There was 0.2 mm precipitation during the 48 h period after spraying and the average maximum temperature during this period was 29.5°C. The trial was of a randomised triplicate design, the sample size per replicate and treatment was 30 bunches. Samples of 60 randomly harvested berries per replicate were taken 3, 6, 9, 24 and 48 h after the sprays. Berries were weighed, immediately deseeded, frozen in liquid nitrogen and stored at −80°C until used. Throughout development weekly samples of 60 randomly harvested berries per replicate were used to measure berry weight and TSS.

In 2012, pre-veraison berries were sprayed twice (16 January 2012, 8 days pre-veraison and 23 January 2012, 1 day pre-veraison) with Control and Ethrel solutions as described above. There was no precipitation during the 48 h period after spraying and the average maximum temperature during this period was 34.5°C. Other changes to the 2012 trial design and sampling regime were as follows: the sample size was increased to 400 bunches per replicate and treatment, samples of 60 berries for deseeding and freezing were taken 1, 6, 24 and 48 h after the initial spray as well as at the developmental stages corresponding to veraison of the Control and veraison of the Ethrel-treated berries and 80 berries per replicates were used for weight and TSS measurements.

For the analysis of developmental changes in gene expression *Vitis vinifera* L. cv Cabernet Sauvignon and Shiraz berries from 3–16 wpf were collected at weekly intervals, (22 November 2010–15 March 2011) from a commercial vineyard (Willunga, South Australia-35°.26, 138°.55) in the 2010/2011 season. Sampling was completed between 09:30 and 11:30, berries (60–200 berries sampled at each time point) were immediately deseeded and tissue frozen in liquid nitrogen and stored at −80°C until used.

For the *ex planta* berry experiment 40 bunches from ten vines (*Vitis vinifera* L. cv Shiraz) were sampled from a vineyard in the Adelaide Hills (Hahndorf, South Australia, -35.018223, 138.838220) 19 days (05 January 2012) before veraison between 09:00 and 10:00 and kept on ice until used.

### Determination of anthocyanin and TSS levels

Individual measurements of TSS (degrees Brix) were taken for the berries sampled from the Ethrel experiments in 2011 and 2012 with an RFM710 digital refractometer (Bellingham Stanley, Tunbridge Wells, UK). TSS analysis and anthocyanin measurements for the berries from the developmental series were done as described by Böttcher et al. [20].

### Phylogenetic analysis

TAR- and YUC-related grapevine sequences were identified by BLASTP searches of the non-redundant NCBI protein database (E-value ≤ 10^-20^) using either the *Arabidopsis* TAA1 or YUC1 protein sequence as the query. NCBI accession numbers for all sequences used in the phylogenetic analysis are listed either in Figure 3 or in Additional file 3. The sequences were aligned using the ClustalW (version 2.0.12) program [80] in the multiple alignment mode and the neighbour-joining unrooted tree was generated with PHYLIP 3.67 [81].

### RNA extraction, cDNA synthesis and qRT-PCR

Total RNA was extracted from grape berry tissue according to Davies and Robinson [82] and further purified as described by Symons et al. [83]. First-strand cDNA for qRT-PCR was synthesized with Transcriptor Reverse Transcriptase (Roche Diagnostics, Mannheim, Germany) using 1 μg of RNA and the Oligo (dT)_18_ primer in a reaction volume of 20 μL following the manufacturer’s instructions. Prior to use cDNA samples were diluted 20-fold. qRT-PCR analyses were performed using the Roche LightCycler 480 SYBR Green I Master kit with 15 μL reactions and a final primer concentration of 0.5 μM. The amplification was performed using the Roche LightCycler 480 system with the following parameters: 5 min at 95°C, 20 s 95°C, 20 s 58°C and 20 s 72°C (45 cycles), 5 min at 72°C, followed by a melt cycle (15 s at 95°C, 45 s at 50°C, continuous heating to 95°C at 0.11°C s^-1^). The gene-specific primer pairs and corresponding accession numbers used for *GH3-1, GH3-2, ACS1, ACO1, ETR2* as well as *ACT2* (reference gene) have been published previously [15,19]. The primers used for putative auxin biosynthesis genes were: for *TAR1* [GenBank: XM_002274511], 5′-CATCTATTCAAGATACTATTGG-3′ and 5′-ATATTGCAGCTACTCTCAAT-3′, for *TAR2* [GenBank: XM_002281372], 5′-CAGCAATGAAGCATATTGAAGG-3′ and 5′-GAGTGAGAGCACCAGGAAATG-3′, for *TAR3* [GenBank: XM_002265837], 5′-CCCAAGATGACTTTGATATGCTG-3′ and 5′-TGATCAACTGATTGTTGATTCCACT-3′, for *TAR4* [GenBank: XM_002266102], 5′-CAGCCTCATCAAGACCCAAGAT-3′ and 5′-TGACGGTTGATTTCATTCTTCG-3′, for *YUC1*[GenBank: XM_002269808], 5′-CAGGAAACTGTCGCAATAGTGG-3′ and 5′-CAAGAACTATGTTGGGTATTGAGAGG-3′, for *YUC2* [GenBank: XM_002269727], 5′-TACACTTTGGAAGCATCACAGC-3′ and 5′-GGTTTGTACTGTGCTGGACTGG-3′ and for *YUC3* [GenBank: XM_002282321], 5′-ATGCCCAAAACGCCATTTCC-3′ and 5′-ATGTCCCGGGCGATATTGAC-3′. Each PCR was performed in triplicate. To calculate the copy number of the genes in each reaction, the purified gene fragments used for the standard curves were quantified using PicoGreen (AGRF, Adelaide, South Australia) and the number of molecules in each standard dilution was determined according to Whelan et al. [84]. The specificity of the reactions was confirmed by melt curve analysis as well as separation on agarose gels and the identity of each product was verified by sequencing (AGRF, Adelaide, South Australia).

### *Ex planta* berry induction assay

Randomly picked berries from the sampled bunches were sterilized in 0.1% (v/v) Chemwet 1000 containing half of a Milton antibacterial tablet L^-1^ (Milton Australia, Laverton North, Victoria) for 10 min and washed three times with sterile nanopure water. All the following procedures were carried out in a laminar flow under sterile conditions. A thin slice was removed from the base of each berry by cutting horizontally through the brush area to facilitate compound absorption and the modified berries were placed on agar. Twenty berries were placed on petri dishes filled with 25 mL of Gamborg’s media, 0.025% (w/v) casein hydrosylate, 0.8% (w/v) agar, pH 5.7-5.8 and one or more of the following additives (final concentrations), respectively: ReTain (125 mg L^-1^ AVG, filter sterilised), Ethrel (72 mg L^-1^ ethephon), 3% (w/v) sucrose. Each plate constituted a replicate and three replicates per treatment and time point were used.

Berries were placed on the plates with the cut surface facing the agar, the plates were sealed with Parafilm and kept in the dark at room temperature. All plates containing Ethrel were stored separately. After 0.5-24 h the berries were harvested, deseeded and frozen in liquid nitrogen.

### Chemical synthesis of labelled IAA-Asp

[Indole-D_5_] IAA-Asp was synthesized as described previously [29] using [indole-D_5_]-labelled IAA as starting substrate.

### LC-ESI-MS/MS analysis of IAA and IAA-Asp

For LC-MS/MS quantification, IAA and conjugates were extracted and quantified from 100 mg of grape berry tissue as described by Böttcher et al. [29].

### Statistical data analysis

The significance of any differences between samples was tested by Student’s *t*-test (unpaired) or ANOVA with Duncan’s *post hoc* test, using IBM SPSS Statistics ver. 20 (IBM Australia, Sydney, NSW, Australia).

## Abbreviations

ACO: 1-aminocyclopropane-1-carboxylate oxidase; ACS: 1-aminocyclopropane-1-carboxylate synthase; AVG: Aminoethoxyvinylglycine; Dps: Days post spray; Dpis: Days post initial spray; CV: veraison of Control fruit; FW: Fresh weight; EV: veraison of Ethrel-treated fruit; GH3: Gretchen Hagen3; Hpis: Hours post initial spray; IAA: Indole-3-acetic acid; TAA: TRYPTOPHAN AMINOTRANSFERASE OF ARABIDOPSIS; TAR: TRYPTOPHAN AMINOTRANSFERASE RELATED; TSS: Total soluble solids; YUC: YUCCA.

## Competing interests

The authors declare that they have no competing interests.

## Authors’ contributions

All authors contributed to the sampling and processing of berry samples derived from field-based and *ex planta* experiments. CB participated in the design of the study, carried out the phylogenetic analyses, primer design and auxin measurements and drafted the manuscript. CAB carried out the qRT-PCR analyses and participated in the auxin extractions. PKB participated in the design of the study and performed the statistical analyses. CD conceived of the study and participated in its design and coordination. All authors read and approved the final manuscript.

## Supplementary Material

Additional file 1 **Transcription of the ethylene biosynthesis genes, *ACS1 *** **and *ACO1*, and the ethylene receptor gene *ETR2 *** **in response to Ethrel. (A)** The expression of *ACS1, ACO1* and *ETR2* in pre-veraison (Shiraz berries from the 2011 trial was analysed by qRT-PCR at the indicated time points after treatment with a Control or Ethrel solution (single treatment (20 days pre-veraison)). Control, dark grey bars; Ethrel, light grey bars. All data represent means ± SE (n = 3). Asterisks indicate significant differences of the mean values of Ethrel-treated samples from the mean values of Control samples as determined with Student’s *t*-test (*p < 0.05, **p < 0.01). **(B)** The expression of *ACS1, ACO1* and *ETR2* in pre-veraison Shiraz berries from the 2012 trial was analysed by qRT-PCR at the indicated time points after treatment with a Control or Ethrel solution (two treatments (8 and 1 day pre-veraison)). Control, dark grey bars; Ethrel, light grey bars. CV, veraison of Control fruit; EV, veraison of Ethrel-treated fruit. All data represent means ± SE (n = 3). Asterisks indicate significant differences of the mean values of Ethrel-treated samples from the mean values of Control samples as determined with Student’s *t*-test (**p < 0.01). (C) The expression of *ACS1, ACO1* and *ETR2,* analysed using qRT-PCR*,* in *ex planta* pre-veraison Shiraz berries exposed to ReTain (125 mg L^-1^ AVG, 3% (w/v) sucrose), Ethrel (72 mg L^-1^ ethephon, 3% (w/v) sucrose), or Control (3% (w/v) sucrose) conditions for the indicated periods of time. Control, dark grey bars; Ethrel, light grey bars; AVG, white bars. Bars represent means ± SE (n = 3) and are denoted by a different letter if the means differ significantly (p < 0.05) using one-way ANOVA followed by Duncan’s post hoc test. Click here for file

Additional file 2 **Expression profiles of selected auxin and ethylene biosynthesis genes throughout Cabernet Sauvignon berry development.** The development of field-grown Cabernet Sauvignon berries was documented by changes in **(A)** TSS, **(B)** berry weight and **(C)** anthocyanin (A520 nm) accumulation. All data represent means ± SE (n = 3). **(D)** Between 3–16 wpf the expression of *TAR1-TAR4*, *YUC1*, *ACS1* and *ACO1* was analysed by qRT-PCR. The expression data are shown for two biological replicates. n.d., not detected. “*v*” indicates veraison as determined by the last time point before a significant increase (ANOVA followed by Duncan’s post hoc test) in TSS levels was recorded. Click here for file

Additional file 3 **GenBank accession numbers of the *Arabidopsis *** **protein sequences used for the phylogenetic analysis.** Click here for file
