# Supplementary figures and images for: Interactions between ethylene and auxin are crucial to the control of grape (Vitis vinifera L.) berry ripening
Source: BMC Plant Biol. 2013 Dec 23;13:222. doi: 10.1186/1471-2229-13-222 (PMC3878033; doi:10.1186/1471-2229-13-222)

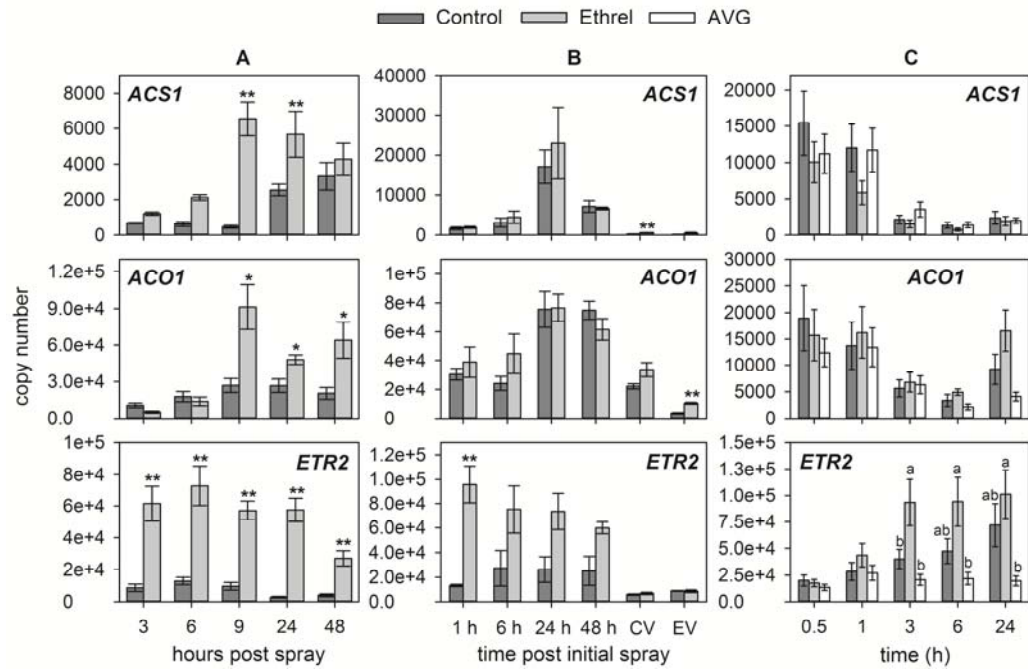

Supplement: Additional file 1 — Transcription of the ethylene biosynthesis genes, ACS1 and ACO1, and the ethylene receptor gene ETR2 in response to Ethrel. (A) The expression of ACS1, ACO1 and ETR2 in pre-veraison (Shiraz berries from the 2011 trial was analysed by qRT-PCR at the indicated time points after treatment with a Control or Ethrel solution (single treatment (20 days pre-veraison)). Control, dark grey bars; Ethrel, light grey bars. All data represent means ± SE (n = 3). Asterisks indicate significant differences of the mean values of Ethrel-treated samples from the mean values of Control samples as determined with Student’s t-test (*p < 0.05, **p < 0.01). (B) The expression of ACS1, ACO1 and ETR2 in pre-veraison Shiraz berries from the 2012 trial was analysed by qRT-PCR at the indicated time points after treatment with a Control or Ethrel solution (two treatments (8 and 1 day pre-veraison)). Control, dark grey bars; Ethrel, light grey bars. CV, veraison of Control fruit; EV, veraison of Ethrel-treated fruit. All data represent means ± SE (n = 3). Asterisks indicate significant differences of the mean values of Ethrel-treated samples from the mean values of Control samples as determined with Student’s t-test (**p < 0.01). (C) The expression of ACS1, ACO1 and ETR2, analysed using qRT-PCR, in ex planta pre-veraison Shiraz berries exposed to ReTain (125 mg L-1 AVG, 3% (w/v) sucrose), Ethrel (72 mg L-1 ethephon, 3% (w/v) sucrose), or Control (3% (w/v) sucrose) conditions for the indicated periods of time. Control, dark grey bars; Ethrel, light grey bars; AVG, white bars. Bars represent means ± SE (n = 3) and are denoted by a different letter if the means differ significantly (p < 0.05) using one-way ANOVA followed by Duncan’s post hoc test. [file 1471-2229-13-222-S1.pdf]

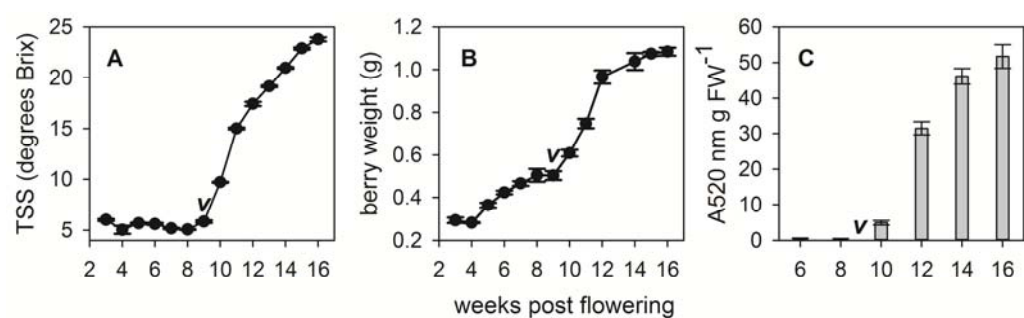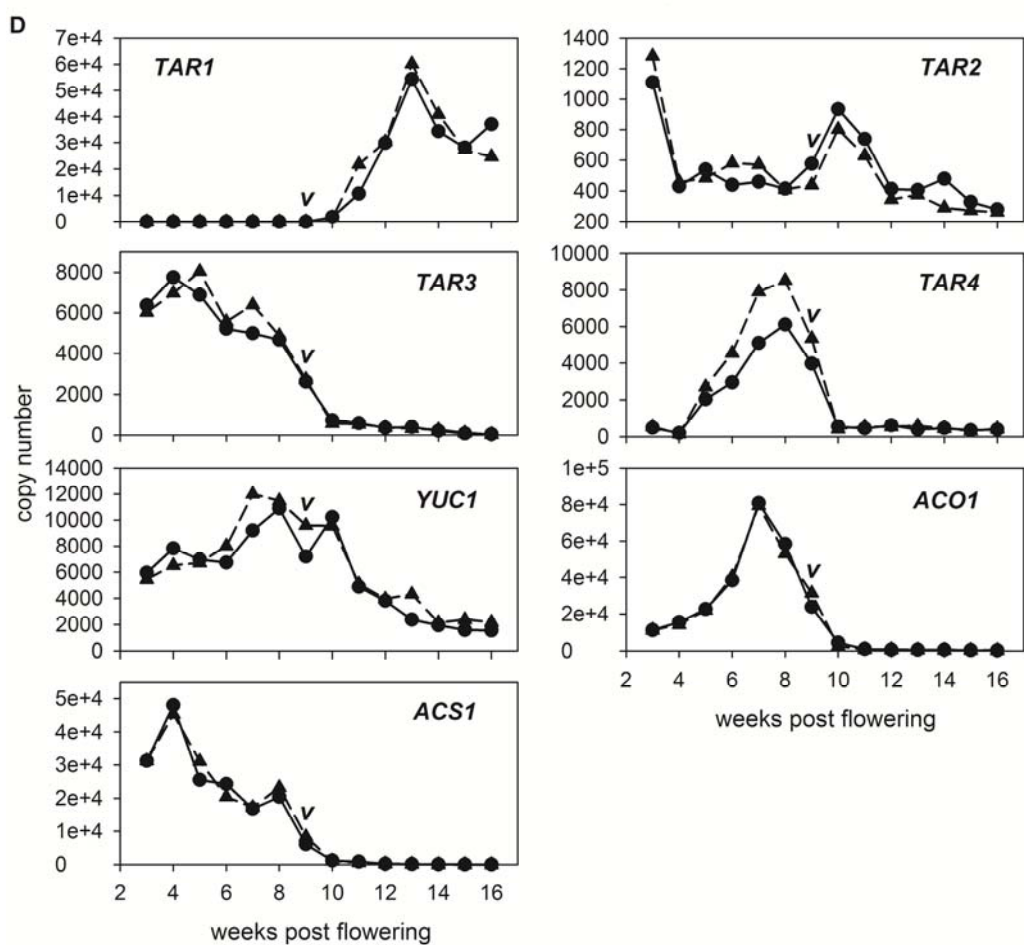

Supplement: Additional file 2 — Expression profiles of selected auxin and ethylene biosynthesis genes throughout Cabernet Sauvignon berry development. The development of field-grown Cabernet Sauvignon berries was documented by changes in (A) TSS, (B) berry weight and (C) anthocyanin (A520 nm) accumulation. All data represent means ± SE (n = 3). (D) Between 3–16 wpf the expression of TAR1-TAR4, YUC1, ACS1 and ACO1 was analysed by qRT-PCR. The expression data are shown for two biological replicates. n.d., not detected. “v” indicates veraison as determined by the last time point before a significant increase (ANOVA followed by Duncan’s post hoc test) in TSS levels was recorded. [file 1471-2229-13-222-S2.pdf]
