# Supplementary material for: Interactions between ethylene and auxin are crucial to the control of grape (Vitis vinifera L.) berry ripening
Source: BMC Plant Biol. 2013 Dec 23;13:222. doi: 10.1186/1471-2229-13-222 (PMC3878033; doi:10.1186/1471-2229-13-222)
Supplement: Additional file 3 — GenBank accession numbers of the Arabidopsis protein sequences used for the phylogenetic analysis. [file 1471-2229-13-222-S3.pdf]

Species	Protein name	Accession no.
Arabidopsis thaliana	AtTAA1	GenBank: AEE30372
	AtTAR1	GenBank: AEE35079
	AtTAR2	GenBank: AEE84940
	AtTAR3	GenBank: AEE31660
	AtTAR4	GenBank: AEE31667
	AtYUC1	GenBank: AEE86075
	AtYUC2	GenBank: AEE83256
	AtYUC3	GenBank: AEE27721
	AtYUC4	GenBank: AED91660
	AtYUC5	GenBank: AED95021
	AtYUC6	GenBank: AED93474
	AtYUC7	GenBank: AEC08802
	AtYUC8	GenBank: AEE85534
	AtYUC9	GenBank: AEE27667
	AtYUC10	GenBank: AEE32365
	AtYUC11	GenBank: AEE30101
